# mTOR signaling in hair follicle and hair diseases: recent progress

**DOI:** 10.3389/fmed.2023.1209439

**Published:** 2023-09-04

**Authors:** Wei Tu, Yu-Wei Cao, Mang Sun, Qian Liu, Heng-Guang Zhao

**Affiliations:** Department of Dermatology, The Second Affiliated Hospital of Chongqing Medical University, Chongqing, China

**Keywords:** mTOR signaling, hair follicle, hair follicle cycles, hair disease, VDR

## Abstract

Mammalian target of rapamycin (mTOR) signaling pathway is a major regulator of cell proliferation and metabolism, playing significant roles in proliferation, apoptosis, inflammation, and illness. More and more evidences showed that the mTOR signaling pathway affects hair follicle circulation and maintains the stability of hair follicle stem cells. mTOR signaling may be a critical cog in Vitamin D receptor (VDR) deficiency-mediated hair follicle damage and degeneration and related alopecia disorders. This review examines the function of mTOR signaling in hair follicles and hair diseases, and talks about the underlying molecular mechanisms that mTOR signaling regulates.

## 1. Introduction

Mammalian target of rapamycin (mTOR) is a serine/threonine protein kinase that detects and combines a range of external and intracellular signals. It also regulates a number of functions, such as gene transcription, autophagy, mRNA translation, protein synthesis, cell proliferation, and metabolism (1, 2). Target of Rapamycin (TOR), named after its inhibitor rapamycin, was first isolated from soil bacteria in the 1970s. Rapamycin, also known as sirolimus, interacts with fk506-binding protein 12 (FKBP12) to limit the function of mTOR (3). Recent studies have demonstrated that mTOR signaling plays a role in a variety of epidermal homeostasis activities, including hair follicle cycles, skin barrier function, and skin healing (4–8). The incidence of numerous hair diseases is linked with dysregulation of mTOR signaling, such as alopecia areata, androgenetic alopecia, fibrosing alopecia, and hair follicle tumors (9–12). In this review, the function of mTOR signaling in hair follicles and hair diseases was highlighted, and underlying molecular mechanisms regulated by mTOR were discussed. We envision that analyzing these mTOR functional implications during hair follicle cycling will be essential for the development of new treatments for hair diseases.

## 2. mTOR signaling

**Figure 1:**
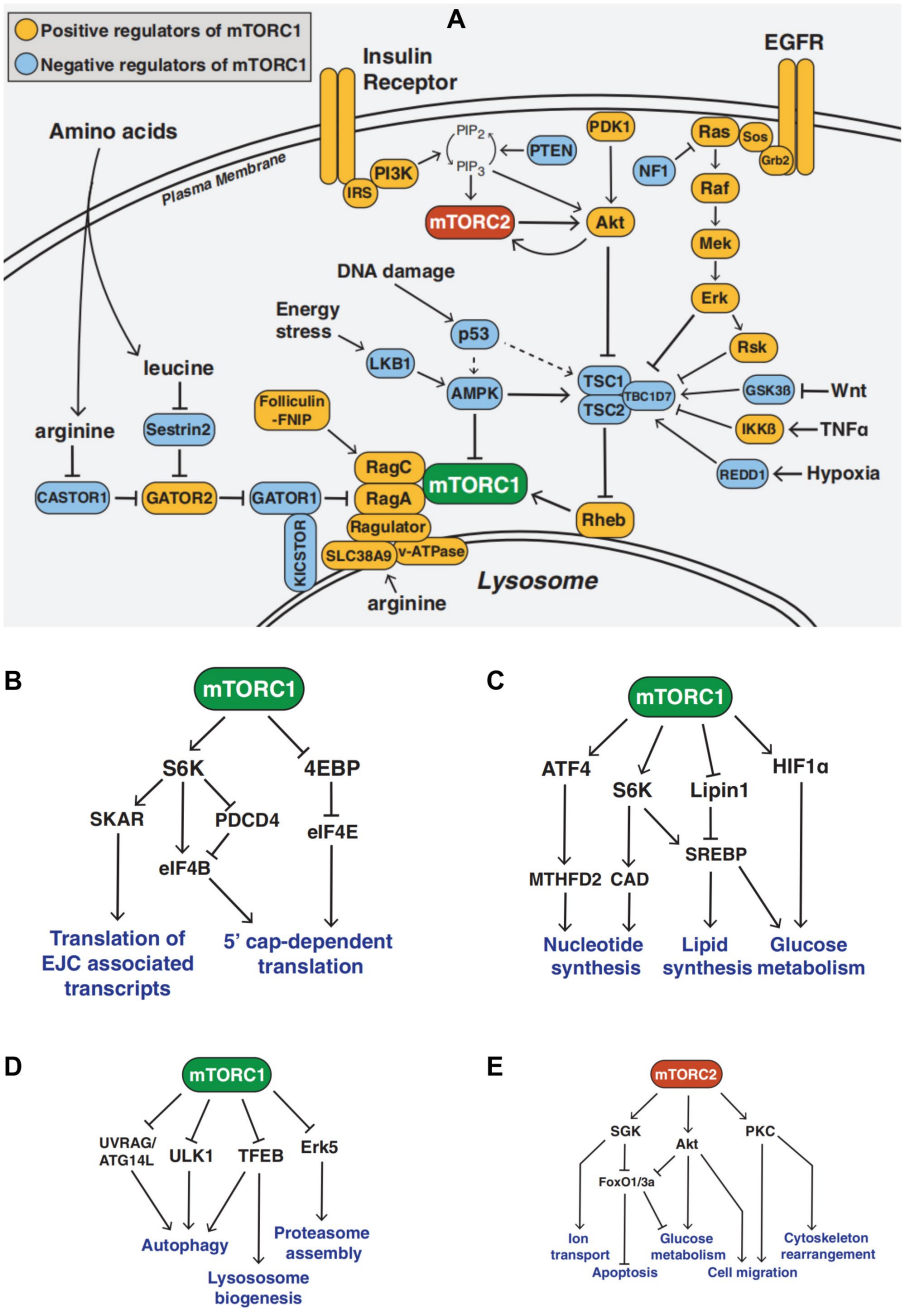
The mTOR Signaling Network **(A)** Overview of the mTOR signaling pathway **(B–D)** mTORC1 signaling in mRNA translation, metabolism, and protein turnover **(E)** mTORC2 signaling function.

The mTOR pathway is a crucial metabolic regulator. The mTOR protein exists in two complexes with distinct structures and functions, called mTOR complex 1 and mTOR complex 2 (1, 13). The proline-rich Akt substrate 40 kDa (PRAS40), an inhibitory subunit, and the regulatory associated protein of mTOR (RAPTOR) are both included in the mTORC1 protein. Numerous growth factor receptors, including the insulin receptor and the epidermal growth factor receptor (EGFR), activate tyrosine kinase adapter molecules at the cell membrane, which attract class I PI3K to the receptor complex. Phosphatidylinositol-3,4,5-trisphosphate (PI [3,4,5] P3), which attracts and stimulates the serine–threonine kinase Akt by phosphorylation on threonine 308, is the pathway through which PI3K activates mTORC1. A major target of Akt is tuberous sclerosis syndrome 2 (TSC2). By phosphorylating TSC2 at threonine 1,462 (Thr1462), Akt prevents the small GTPase RAS homolog enriched in the brain (Rheb) from acting as a GTPase-activating protein (GAP), keeping it in a GTP-bound state and activating mTORC1. The activation of class I PI3K ultimately leads to the activation of mTORC1 by inhibiting TSC2. mTORC1 is activated in organelles like peroxisomes and lysosomes (14). Full activation of mTORC1 requires many nutrients and energy sources, such as glucose, lipids, oxygen, leucine, arginine, and a high ATP/AMP ratio (15, 16) (Figure 1A).

The functions of mTORC1 include mRNA translation, metabolism, protein turnover, etc. mRNA translation includes translation of the multi-protein exon junction complex (EJC)-associated transcripts and 5′ cap-dependent translation (17, 18). Metabolism includes nucleotide synthesis, lipid synthesis, and glucose metabolism (19). Protein turnover includes autophagy, lysosome biogenesis, and proteasome assembly (20, 21). Activated mTORC1 can activate s6k, promote protein synthesis, inhibit 4E-BP, regulate translation initiation, phosphorylate ULK1 and ATG, and inhibit the occurrence and development of autophagy. Additionally, mTORC1 inhibition increases ULK1 kinase activity to encourage autophagy (22) (Figures 1B–D).

Rapamycin-insensitive mTOR companion (RICTOR) and mammalian stress-activated Map kinase interacting 1 (mSIN1) are both found in mTORC2. Additionally, PI3K through PI (3–5) p3 activates mTORC2, which phosphorylates Akt on serine 473. The functions of mTORC2 include ion transport, cell migration, apoptosis, glucose metabolism, and cytoskeleton reorganization (23–26). mTORC2 acts downstream of growth factor signaling, activates Akt, PKC, and Sgk1, and stimulates cell growth, proliferation, survival, and cytoskeletal remodeling (27–29). In certain cells and in living animals, chronic rapamycin administration reduces mTORC2 signaling (30, 31) (Figure 1E).

## 3. Roles of mTOR signaling in hair follicle development and hair regeneration

Hair is a fiber of amazing tensile strength composed primarily of terminally differentiated, dead keratinocytes. The hair follicle is an important structure supporting hair growth, and its periodic structural changes are the basis of hair cycles. The disease and aging of hair follicles will lead to an imbalance of hair cycles, and eventually cause hair loss and other hair diseases. Hair follicles generally go through cycles of growth (anagen), regression (catagen), and rest (telogen) (32). The most active stage of hair follicle development is known as the anagen stage, during which the hair grows quickly and completely forms a hair shaft. Hair follicles typically enter catagen when the hair shaft stops developing, the capacity of cells to proliferate and differentiate starts to wane, cells start to experience apoptosis, and the hair follicles disintegrate quickly. The hair follicle enters telogen after the catagen stage, which is characterized by the lowest biological activity of the hair follicle and the loss of the hair shaft. The development of new hair shafts, the loss of old hair, and changes in dermal papillae structure and morphology are the key changes that take place during the hair cycle (33). Hair follicle stem cells (HFSCs) in the bulge control these transitions of growth cycles (34). HFSCs are mostly in a quiescent state, but they also periodically undergo activities such as cell migration, proliferation, and differentiation (35, 36).

### 3.1. mTOR signaling in hair follicle cycles and hair follicle stem cells

The regulation mechanism of HFSCs and HF cycles involves the mTOR pathway, the BMP pathway, and the Wnt pathway (37–39). Accumulating evidence suggests mTOR signaling is vitally involved in hair follicle cycles and the stabilization of hair follicle stem cells. Hair cycle beginning was delayed in a pharmacological test *in vivo* using the particular mTORC1 inhibitor, rapamycin, demonstrating that mTORC1 has a role in anagen entrance. The timing of the anagen beginning phase of the hair cycle may be regulated by mTORC1 (39). The balance between HFSC quiescence and activation during hair regeneration is controlled by mTOR signaling, which is a key regulator in this process. When HFSCs are active at the telogen-to-anagen transition, mTORC1 signaling is also stimulated in these cells. The activation of the HFSC is noticeably delayed and the telogen phase is prolonged in HFs that are unable to react to mTOR signaling. The mTORC1 signaling pathway negatively affects BMP signaling and balances BMP-mediated inhibition of anagen initiation, ultimately promoting HFSC activation and hair growth (40). Interfering with miR-27a causes PIK3R3 to express more, which in turn causes AKT and MTOR to express and be activated more. The proliferation and decreased apoptosis of HFSCs were both boosted by the activation of mTOR signaling (41).

The excessive activation of mTOR was specifically linked to both HF hyperproliferation and HFSC exhaustion. As import signaling pathways, TGF-β adjusts the Akt/mTOR pathways through the Smad2/3 (42). A significant downstream element of the pathway *via* which Wnt1 stimulation can cause cell proliferation and tissue aging is the mTOR protein. By blocking GSK3, Wnt lessens TSC2’s inhibitory impact on mTOR, enhancing mTOR activity. Wnt1 expression causes HF cells to hyper proliferate and rapidly exhaust their CD34^+^ stem cells, as shown by the ablation of HF stem cells, which also occurs at the same time as the activation of cell senescence pathways. Rapamycin prevented Wnt1-expressing mice from losing CD34^+^ stem cells in the bulge area of their hair follicles (43).

A remarkable characteristic of healthy anagen stage hair follicles is the relative immune privilege (IP) that their epithelium displays from the bulge area, the habitat for HF stem cells, downstream to the hair bulb (44). Alopecia areata (AA) and primary cicatricial alopecia (PCA) are the results of an immune attack on the HF when this IP collapses, which is caused by inflammatory infiltrates that collect around the bulge and bulb (45). In the lower follicle ORS and bulb of the hair follicles in the lesional scalps of PCA patients, p-mTOR and p-p70S6K expression rose, whereas p-4EBP1 expression dropped (12). The immunogenicity of differentiated stem cells was markedly reduced by the suppression of mTOR signal pathways (46). These imply that IP collapse may be associated with aberrant mTOR activation in the bulb of the hair follicle. Isolated immune cells are sometimes seen in and around the bulb of an anagen stage HF during the pathological hair development cycle. Dendritic cells, mast cells, NK cells, CD4^+^T and CD8^+^T cells, and other inflammatory cells are present in the perifollicular infiltration. Dendritic cells and macrophages are frequently the second kind of cell to enter intra-follicular sites after CD8^+^T cells. IFN-γ and Substance P, the two known HF IP collapse inducers, are active in both animal and people (44). Inhibition of mTOR signaling reduces the function of many immune cells and may contribute to the maintenance of IP in the hair follicle. mTOR inhibition caused immunosuppression. Damage to the PI3K intracellular signal-transmitting enzymes led to phenotypic abnormalities, a substantial decrease in the number of NK cells in peripheral organs, and decreased cytokine release in other cells (47). Both TGF-β and the mTOR inhibitor rapamycin decreased NK cells’ metabolic activity, proliferation, and abundance of several NK cell receptors, as well as their capacity to carry out cytotoxic action (48). Rapamycin can decrease the proportion and cytotoxic function of CD4^+^ cytotoxic T lymphocytes (CTLs) in Graves’ orbitopathy (49). When the AKT/mTOR pathway was suppressed, the increases in cytotoxic molecules caused by VEGF-A were considerably decreased. VEGF-A treatment activated the AKT/mTOR pathway in CD4^+^ CTLs (50). To maintain glucose absorption and glycolysis in CD8^+^ T cells, mTORC1 activity is essential. The mTORC1-HIF1 pathway, which effectively connects mTORC1 to a multitude of transcriptional processes, is required for effector CTLs to sustain glycolysis and glucose metabolism (51).

Inhibition of mTOR signaling can increase regulatory T cells (Tregs) number, induce immune tolerance, and reduce inflammation, which may help reduce the number of local inflammatory cells around the bulge and bulb and maintain relative IP. As a distinct marker for Tregs, the fork head transcription factor (Foxp3) was discovered. Only T cells expressing Foxp3 were able to prevent transplant rejection *in vivo* after being exposed to antigen in the presence of TGF-β *in vitro*, in contrast to their Foxp3-negative counterparts (52). The ratio of Treg to T effector cells (Teff) and the response of mTOR to a variety of microenvironmental stimuli determine whether an individual is tolerant of something or inflammatory. The balance between the quantity of Tregs and Teff is increasingly thought to determine whether the result of an immune response is one of tolerance or inflammation. Activating the GCN2 pathway had no effect on the activation of Foxp3 in naïve CD4^+^ T cells in the presence of modest doses of TGF-β, while inhibiting the mTOR pathway with rapamycin increased Foxp3 expression. Metabolism and Foxp3 expression are regulated by nutrient and environmental sensing *via* mTOR. For controlling cell metabolism and Foxp3 expression, mTOR functions as an integrator of signals that come from a variety of cell surface receptors and nutrient-sensing pathways (53).

**Figure 2:**
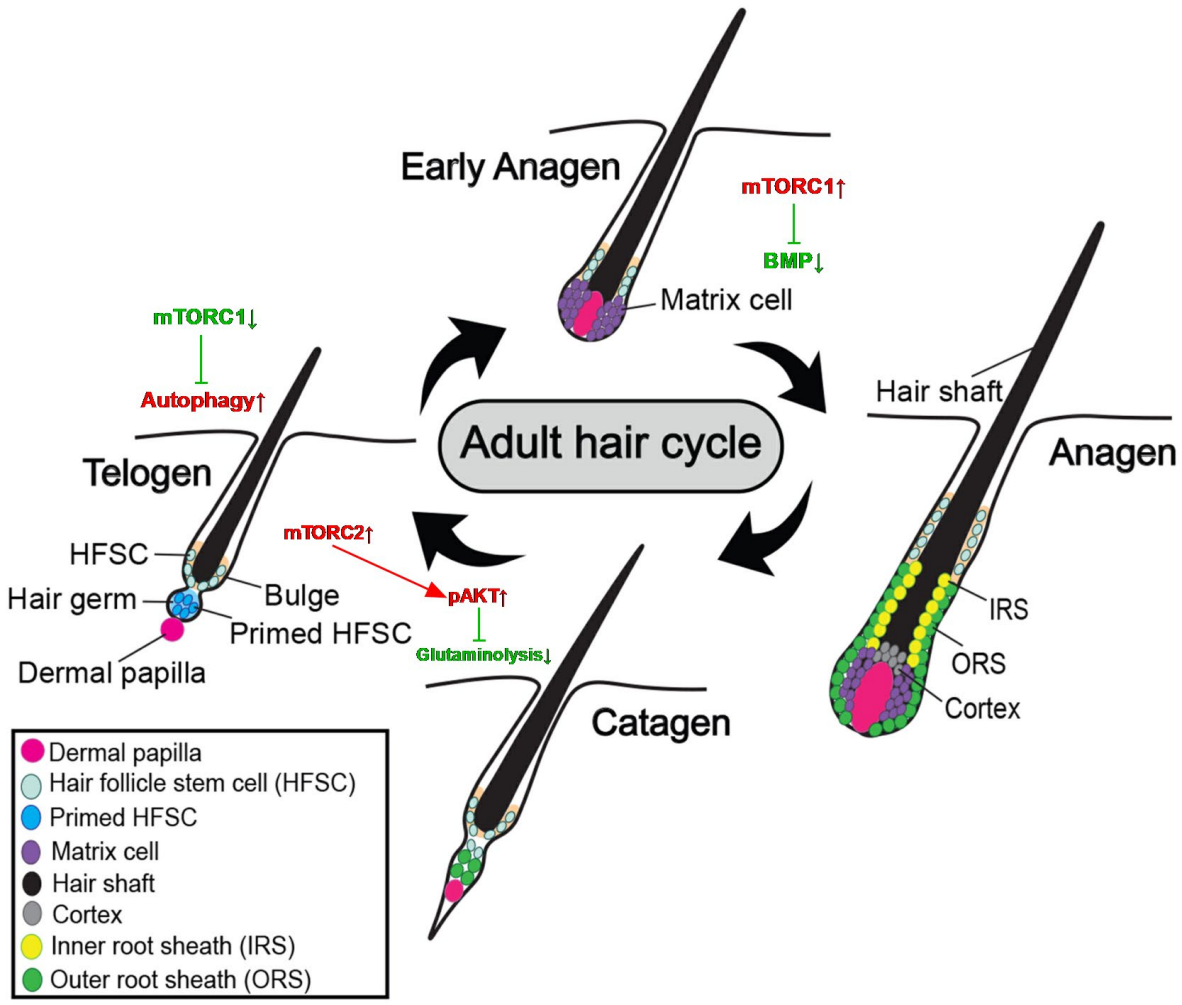
Roles of mTOR signaling in hair follicle development and regeneration.

Inhibition of glutamine metabolism by mTORC2 signaling contributes to progenitor reversibility and reestablishes HFSC niche function (6). Oxidative phosphorylation is activated, and glutamine enters the TCA cycles during the shift from HFSCs to the ORS progenitor state. The HFSC niche is hypoxic, and low pO_2_ (the partial pressure of oxygen) promotes a stem cell state. Low pO_2_ inhibits the expression of glutaminase in a way that is reliant on mTORC2-Akt, thereby reducing glutamine hydroxylation to stop the glutamate from entering the Krebs cycles and promoting the return of progenitor cells to the HFSC state, thereby allowing progenitor cells to return to the low oxygen niche, restore stem cell status, and restore bulge to maintain hair follicle stem cells throughout the long run. Loss of mTORC2 impairs niche regeneration of progenitor cells and triggers hair follicle stem cell failure, and for HFSC destiny reversibility and long-term maintenance *in vivo*, mTORC2 is necessary (Figure 2).

### 3.2. Factors affecting hair growth by regulating mTOR signaling

Autophagy, heat stress, and Vitamin D are three factors associated with mTOR signaling that play a role in alopecia. Autophagy refers to the starvation-induced degradation of cytoplasmic components under catabolic conditions with limited nutrient supply and is critical for providing substrates for energy production and clearing damaged organelles (54). mTOR signaling is necessary for the activation of hair follicle stem cells and their entry into anagen (39, 40, 43). However, research has also reported that inhibiting mTOR signaling in quiescent telogen hair follicles can activate autophagy and stimulate telogen hair follicles to initiate the anagen phase and accelerate hair regrowth. The metabolites a-ketoglutarate (a-KG) and rapamycin are examples of small compounds that may inhibit mTOR and activate autophagy. Autophagy is also boosted during the anagen phase of the natural hair follicle cycle (55). Activation of autophagy may help to rescue alopecia caused by a shortened anagen phase, a prolonged dormant phase, and/or blocked anagen induction (55). Autophagic activity is inhibited in the HF of AA mice. Compared to untreated AA animals, autophagy induction reduced the severity of AA, but autophagy blocker therapy increased illness (56). Intrafollicular autophagy is crucial for HF growth and plays a fundamental role in HF anagen maintenance (57). Hair matrix keratinocytes of organ-cultured HFs exhibit an active autophagic flux in anagen, but it changes once catagen begins, according to analysis of endogenous lipidated Light Chain 3B and sequestosome 1 proteins as well as ultrastructural visualization of autophagosomes at all stages of the autophagy process. The anagen hair matrix exhibits early catagen and increased keratinocyte death when follicular autophagy is genetically inhibited, suggesting that autophagic flux in this matrix is essential for sustaining this state. Moreover, a hair loss therapy greatly boosts intrafollicular autophagy, which lengthens the anagen phase of human hair development. Intrafollicular autophagy is eminently targetable for therapeutic regulation of human hair growth (57).

The effect of heat stress on phosphorylated mammalian target of rapamycin (p-mTOR) expression occurs in a tissue-specific manner. Heat stress stimulates the mTOR signaling pathway in skeletal muscle, but significantly inhibits the expression of the p-mTOR protein in hair follicle cells and regulates the development of hair follicle cells. By increasing the expression of BMP2 and BMP4, decreasing the mRNA levels of noggin, IGF1, and IGF1R, and increasing the protein level of p-mTOR, heat stress decreased the number of hair follicles (58). Hair follicle mTORC1 signaling can be activated after radiation injury to promote hair follicle regeneration and hair growth (59). By facilitating the activity of the Akt/mTOR signaling pathway and mediating the activation of HFSCs, LGR4 governs the advancement of the hair cycle. The absence of LGR4 prevents the activation of the Akt/mTOR signaling pathway in the HF cycle. In Lgr4 mutant mice, reactivation of Akt signaling reversed its delayed HF cycle, suggesting that Lgr4-regulated HF homeostasis and HFSC activation depend on the activity of the mTOR signaling pathway (60). Micro-current electrical stimulation can increase various growth factors in human hair follicle papilla cells and mouse hair follicles, activate the PI3K/AKT/mTOR pathway, and Wnt/β-catenin pathway significantly, and promote hair growth (61).

**Figure 3:**
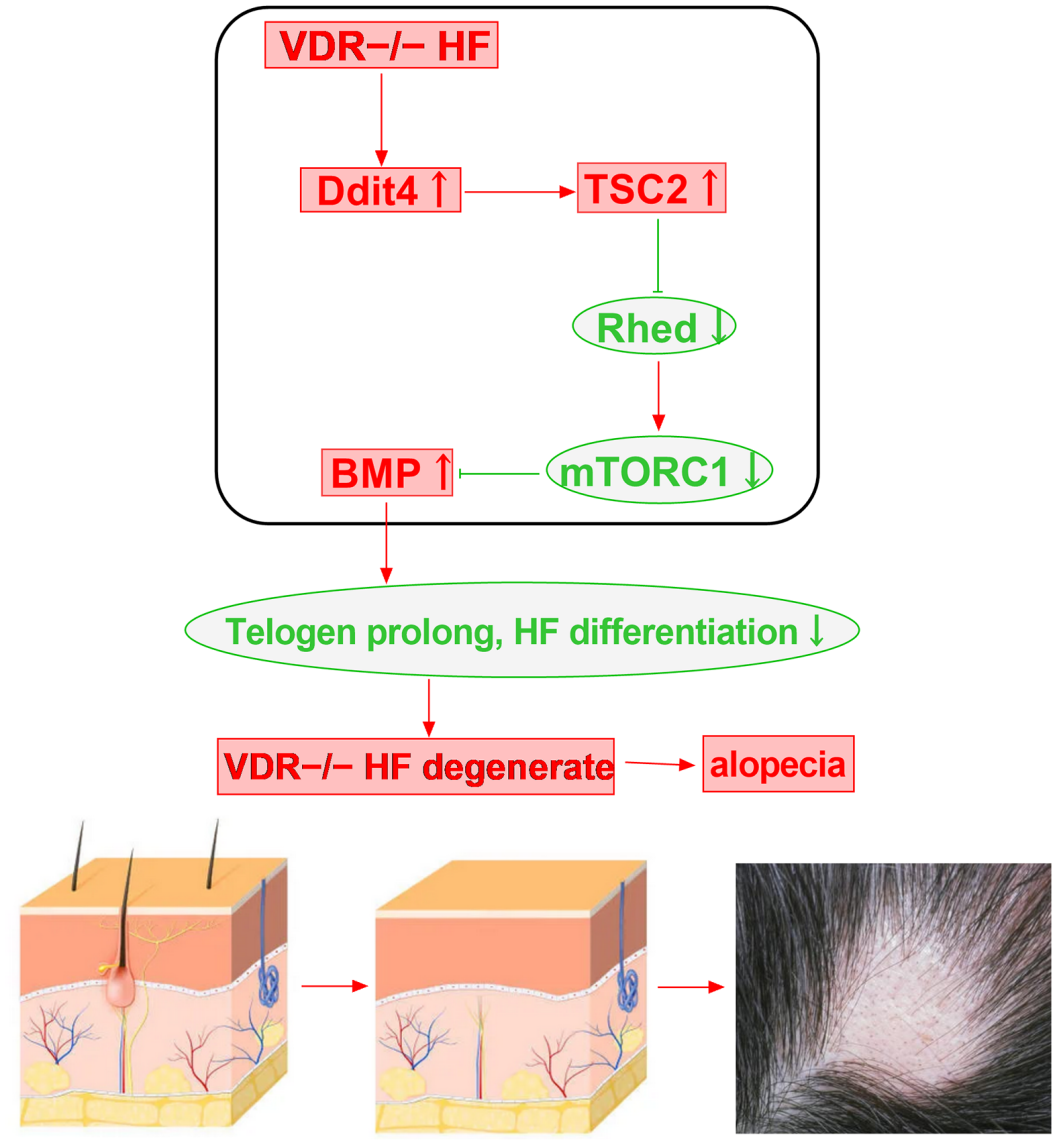
Vitamin D receptor deficiency may induce alopecia through the Ddit4/mTOR signaling pathway.

Hair follicle damage and degeneration may have a great correlation with mTOR signaling in Vitamin D receptor (VDR) knockout mice. The follicular epithelium of VDR−/− mice is defective in the regression-to-rest morphogenetic stage. VDR−/− hair follicles exhibit DNA damage-inducible transcript 4 (Ddit4) stress compartments with increased Ddit4 during morphogenesis. In VDR−/− mice, abnormalities in Ddit4 signaling impact follicular integrity by disrupting follicular energy balance. In epidermal keratinocytes, the mTOR inhibitor Ddit4 is a direct transcriptional target of the VDR. Ddit4 suppresses mTOR signaling by activating TSC2, a GTP hydrolase activating protein, which subsequently prompts Rheb to inactivate mTOR in a GDP-bound form. In VDR-deficient hair follicles, mTOR signaling is reduced, BMP signaling is increased, the transition from catagen to telogen phase is advanced and prolonged, hair follicle cell differentiation is reduced, and eventually the hair follicle degenerates, which can cause various hair loss diseases (Figure 3) (62).

## 4. Role of VDR in hair disease

mTOR signaling is an important component of maintaining hair follicle circulation and hair follicle stem cell stability. Once mTOR signaling is out of balance or missing, it can lead to the occurrence of various hair follicle and hair diseases. In addition, 1,25D_3_ affects mTOR function in the cytoplasm in multiple ways (63), The vitamin D receptor and Ddit4 are important regulators of mTOR signaling and play crucial roles in hair loss diseases.

Alopecia areata is a frequent kind of hair loss that is characterized by well-defined, skin-colored, round to oval, non-scarring patches. Many studies have reported that serum 1,25D_3_ levels in patients with alopecia areata are lower than those in healthy subjects, some of which are accompanied by compensatory increases in parathyroid hormone levels, and that serum 1,25D_3_ levels less than 30 ng/ml are associated with the occurrence of alopecia areata (64, 65). In individuals with alopecia areata, vitamin D insufficiency is adversely correlated with the severity and duration of the condition. The tissue and serum VDR levels of patients with alopecia areata were significantly lower than those in the control group. In comparison to healthy skin, alopecia lesions had considerably reduced levels of VDR expression in the hair follicles and epidermis, and the degree of alopecia areata was significantly negatively correlated with VDR in tissues (66). However, another study revealed that reduced VDR expression in alopecia areata was inversely correlated with hair follicle histological inflammation but not with serum vitamin D levels, disease severity, pattern, or duration (10). By modulating mTOR signaling, vitamin D and VDR deficiency may exacerbate alopecia areata and cause severe hair loss. Downregulation of VDR in hair follicles is related to lower hair development.

The most prevalent kind of progressive hair loss is androgenetic alopecia (AGA), which is also known as androgen-related progressive hair thinning. AGA is further classified into male-and female-pattern hair loss. Many studies have confirmed that the serum vitamin D level of AGA patients is lower than that of the control group and that vitamin D deficiency exists (67, 68). AGA patients with high levels of dihydrotestosterone are deficient in all vitamins and trace minerals, including vitamin D, zinc, copper, magnesium, selenium, and vitamin B12 (69). Serum vitamin D levels can be used as an index to judge the incidence and severity of AGA. In addition, the levels of VDR in the scalp and blood of AGA patients were also significantly lower than normal (70). Hair loss occurs in patients with an inherited VDR deficiency, and VDR knockout mice are unable to initiate a new hair cycle. Many studies have found that the serum 1,25D_3_ concentration in female pattern hair loss patients is lower than that in the control group (71, 72). The average serum 1,25D_3_ concentration has nothing to do with the course of disease or age, but there is a certain correlation with the severity of the disease. Some studies believe that women with a family history of female pattern hair loss and vitamin D lacking or insufficiency are more likely to suffer from female pattern hair loss, but some studies do not support this view (71). The serum and tissue VDR levels of female patients were higher than those of male patients. Compared with healthy controls, the serum and tissue VDR concentrations of female pattern hair loss patients were significantly lower, but they were not related to the severity of the disease (70). In the study of the correlation between VDR gene polymorphisms (Cdx-1 and Taq-1) and female pattern hair loss, it was found that Taq-1 and Cdx-1 may be risk factors and play a role in the duration of female pattern hair loss (73). Vitamin D and VDR are so closely related to AGA that they may regulate the process of AGA through the Ddit4/mTOR pathway.

## 5. Roles of mTOR signaling in hair disease

Alopecia areata (AA) and primary cicatricial alopecia (PCA) are the results of an immune attack on the HF when this IP collapses. The relationship between the PI3K/Akt/mTOR pathways and the molecular markers that are specific to AA, such as NK cells, CD4^+^ and CD8^+^, MHC classes I and II cells, and migration inhibitory factor (MIF), is indicative of the function that these pathways play in the disease. Immunological mediators such macrophages, Langerhans’ cells, and cytokines, as well as CD4^+^, CD8^+^, and other immunological mediators, have all been linked to the onset and progression of AA (74–76). CD8^+^ T cells, NKG2D^+^ cells, IFN-γ, and IL-15 have a significant role in the etiology of AA. Since both IFN-γ and IL-15 signal *via* the Janus kinase (JAK) pathway, JAK inhibitors are promising drugs for managing AA *in vivo* (77). JAK1 and JAK3 selective inhibitors effectively stimulated hair growth and reduced AA-related inflammation. But a number of JAK2-selective inhibitors failed to reinstate hair growth. The effectiveness of this therapy in reversing autoimmune illnesses like AA may be explained mechanistically by the targeted production of T cell exhaustion utilizing a JAK1/3 inhibitor (78, 79). By promoting the growth of alopecic T cells, exogenous IL-7 hastened the start of AA. In contrast, IL-7 inhibition prevented AA from progressing and reversed early AA in C3H/HeJ mice. The overall number of most T cell subsets was significantly decreased by IL-7R blockage, while regulatory T cells (Tregs) were somewhat spared (80). It was confirmed that the AA risk genes IL2RA, STX17, and TNXB are regulated by miR-30b at particular sites. This study suggests that microRNAs have a role in the development of AA (81). CXCR3-blocking antibodies can stop the development of AA in the graft model and prevent the buildup of CD8^+^T cells in the skin (82). The care of HF diseases linked to redox imbalance, such as HF graying and HF ageing, androgenetic alopecia and alopecia areata, may benefit from small molecule NRF2 activators. The reduction in early catagen and hair growth inhibition brought on by oxidative stress was decreased by Nrf2 pre-activation, which also reversed the reduction in hair matrix proliferation brought on by reactive oxygen species (83, 84). γδT-cells can cause early catagen, dystrophy, and HF immune privilege breakdown, which become the main reasons for AA (Table 1) (85, 86).

**Table 1:** Summary of the treatment of hair diseases.

Hair diseases	Routine treatment	Future treatment strategies
AA	Corticosteroids, Minoxidil, etc.	VDR, Ddit4/mTOR pathway
	Physical therapies:	JAK1/3 inhibitors (IFN-γ, and IL-15)
	Laser therapy	γδT-cells, CD8^+^T cells
	Micro-needling	IL-7 inhibitors
		miR-30b
		CXCR3 blockade
		NRF2 activators
PCA (LPP, FFA)	Corticosteroids, Minoxidil	mTOR inhibitors
	Tacrolimus, etc.	JAK inhibitors
	Laser therapy	mast cells, CD8^+^T cells
		PPAR-γ
AGA	Finasteride, Minoxidil, etc.	VDR, Ddit4/mTOR pathway
	Physical therapies:	PPAR-γ
	Micro-needling	NRF2 activators
	Laser therapy	mTOR signaling

As a type of PCA, Lichen planopilaris (LPP) includes the classical form, frontal fibrosing alopecia (FFA), and Graham-Little syndrome. LPP and FFA can lead to permanent alopecia, which is considered to be related to hair follicle stem cell damage and the mTOR signaling pathway. The mTOR signaling pathway protein is found in all regions of healthy hair follicles, but it has abnormal expression in those of lichen planopilaris and frontal fibrosing alopecia patients. The expression of p-mTOR decreased in the interfollicular epidermis of the patients’ skin lesions, and increased in the lower part of the hair follicle in the ORS and the hair follicle bulb (12). It has been demonstrated that JAK inhibitors are useful for treating LPP (87). In PCA lesions, immune-histological investigations found that the number of mast cells (MC) was elevated. Gene expression analysis identified common PCA-related pathways, particularly those strongly connected with MC (88). Peroxisome proliferator-activated receptor (PPAR)-γ-mediated signaling plays an important role in the LPP/FFA associated with IP collapse (89). Due to its ability to reduce T-lymphocyte activation, proliferation, and antibody generation, sirolimus is another possible therapy for FFA (90).

AGA is distinguished by androgen-related, progressive hair loss in a specific pattern. By reducing the activity of the enzymes 17, 20-lyase of cytochrome P450c17, and 3-hydroxysteroid dehydrogenase, PPAR-γ signaling impacts androgen production, which lowers androgen levels. These results warrant investigating PPAR-γ antagonist therapy for the management of AGA (91). Small molecule NRF2 activators may be helpful in the treatment of HF illnesses including androgenetic alopecia and alopecia areata that are connected to redox imbalance (84). Dihydrotestosterone (DHT) interacts with the dermal papilla cells (DPCs), which line the base of the hair follicle, to cause AGA. The suppression of the hair development cycle in immortalized DPCs may be lessened by DHT’s stimulation of mTOR (92). By triggering the TGF-β signaling pathway, *Serenoa repens* extracts encourage hair growth and repair in AGA mice models (93). Regenerative stem cell treatment for androgenic alopecia may occur through the Wnt/β-catenin pathway (94).

The mTOR pathway is crucial for treating hair disorders and issues. In rat dermal papilla cells, limonin can promote anagen signaling by activating autophagy by targeting the Wnt/β-catenin and/or PI3K/AKT pathways, suggesting a possible nutrient for hair loss therapy (95). The effect of *Eclipta prostrata* on promoting hair follicle and hair growth is achieved by increasing the expression of FGF-7 and FGF-5 in human dermal papilla cells and activating the mTOR signaling pathway (96). The molecular mechanism of adenosine promoting hair growth includes regulating the activity of Gsk3β to activate adenosine receptors to mimic the Wnt/β-catenin signaling pathway, Nevertheless, PKA and mTOR activity are necessary for the route to be activated. The Wnt/β-catenin pathway is activated by adenosine mostly through the Gas/cAMP/PKA/mTOR cascade (97). Myristoleic acid induces autophagosome formation by reducing the levels of p-mTOR, Atg7, and LC3II, promoting anagen phase signaling through autophagy and cell cycle progression, which may be useful for alleviating hair loss (98). kin barrier integrity and T cell homeostasis are compromised by RNA stress brought on by SKIV2L loss, which promotes mTORC1 signaling in keratinocytes and T cells. Epidermal hyperplasia and abnormalities in hair formation are caused by the skin-specific deletion of Skiv2l, including skin lesions and hair fragility with nodular alopecia in the hair-hepatic syndrome. Using the mTOR inhibitor rapamycin to treat skiv2l-deficient mice alleviated the cutaneous symptoms that were present (99). Two-pore channels are a class of non-selective cation channels in the lysosome system. ATP can block the activation of two-pore channels (TPCs) through mTOR, resulting in the loss of TPCs, which can affect angiogenesis, autophagy, and human hair pigmentation (100). FLCN is required for the recruitment of mTORC1 to lysosomes upon amino acid stimulation. FLCN gene deficiency can lead to Birt-Hogg-Dubé syndrome, including fibrofolliculoma, lung disease, and kidney disease. Fibrofolliculoma is related to FLCN and mTOR signaling (101). Cronkhite-Canada syndrome can induce alopecia, which may be benefited by the incorporation of sirolimus (102).

## 6. Summary and remaining problems

The transition of hair follicles between different phases of the hair cycle is regulated by the balance between growth-stimulatory and inhibitory signals, mainly through the mTOR pathway. mTORC1 and mTORC2 exhibit corresponding functions in controlling hair follicle cycles and maintaining the HFSC pool. The activation of mTORC1 is necessary for the activation and entry of hair follicle stem cells into anagen, while the activation of mTORC2 is very important for the maintenance of HFSC and helps the progenitor cells return to the quiescent HFSC state. The mTORC1 signaling pathway balances BMP-mediated inhibition of anagen initiation, ultimately promoting HFSC activation and hair growth. Inhibition of mTOR signaling in quiescent telogen hair follicles can activate autophagy and stimulate telogen hair follicles to initiate the anagen phase and accelerate hair regrowth. VDR−/− hair follicles can up-regulate Ddit4 signaling and down-regulate mTORC1 signaling, then prolong the transition from catagen to telogen, reduce hair follicle cell differentiation, and eventually induce hair follicle degeneration and hair loss. mTOR signaling may be a critical cog signaling in VDR deficiency-mediated hair follicle damage and degeneration and related alopecia disorders such as alopecia areata, androgenetic alopecia, lichen planopilaris, frontal fibrosing alopecia, and even hair repigmentation. The precise interaction between mTOR and other regulating pathways such as BMP and Wnt in hair follicles requires further study. Inhibition of mTOR signaling in quiescent telogen hair follicles can initiate hair growth, but mTOR signaling is vital in anagen and catagen hair follicles for hair follicle cycles and HFSC pool maintenance. How to rationally utilize mTOR signaling to promote hair growth at different growth stages seems worthy of further study.

The etiology of alopecia areata and primary immune alopecia is related to the destruction of immune privilege in the hair follicle bulb and bulge, which leads to the up-regulation of local MHC classes I and II receptors and the accumulation of CD8^+^ T cells, NK cells, and other immune cells, eventually leading to the occurrence of alopecia. Inhibition of mTOR can induce immunosuppression and immune tolerance, reduce the immunogenicity of hair follicle stem cells, reduce the number of local immune cells, and reduce the function of immune cells, which may help to maintain immune privilege in hair follicles and relieve the clinical symptoms of AA and PCA etiologically. As import signaling pathways of mTOR, Wnt and TGF-β pathways play a critical role in AGA. Therefore, mTOR targeting would be an improvement to the current treatment regimen.

## Author contributions

WT created the plot of the manuscript and first draft with the help of Y-WC, MS, and QL. H-GZ critically edited and revised the manuscript. All authors have read and agreed to the published version of the manuscript.

## Funding

This work was funded by the National Natural Science Foundation of China for H-GZ (no. 82173440).

## Conflict of interest

The authors declare that the research was conducted in the absence of any commercial or financial relationships that could be construed as a potential conflict of interest.

## Publisher’s note

All claims expressed in this article are solely those of the authors and do not necessarily represent those of their affiliated organizations, or those of the publisher, the editors and the reviewers. Any product that may be evaluated in this article, or claim that may be made by its manufacturer, is not guaranteed or endorsed by the publisher.
